# The value of cough sound monitoring via an audio-enabled smartwatch for OSA screening in COPD patients: a cross-sectional exploratory study

**DOI:** 10.3389/fmed.2025.1650014

**Published:** 2025-10-08

**Authors:** Cheng Zhang, Chunbo Zhang, Zhe Jin, Kunyao Yu, Shanshan Wei, Meng Zhang, Zhou Jin, Jiping Liao, Guangfa Wang

**Affiliations:** Department of Respiratory and Critical Care Medicine, Peking University First Hospital, Beijing, China

**Keywords:** cough sound, smartwatch, COPD, OSA, audio

## Abstract

**Objective:**

The purpose of this study is to explore the value of cough sounds and forced exhalation sounds monitored by smartwatches with audio collection capabilities for screening obstructive sleep apnea (OSA) in patients with chronic obstructive pulmonary disease (COPD).

**Methods:**

Stable COPD patients were recruited from an outpatient clinic. All participants completed questionnaires and underwent pulmonary function testing and overnight polysomnography (PSG). A novel smartwatch capable of collecting audio signals was worn to continuously monitor peripheral oxygen saturation (SpO₂), heart rate (HR), heart rate variability (HRV), and respiratory rate (RR). Additionally, voluntary cough and forced exhalation sounds were recorded twice daily. Audio data were denoised, segmented, and analyzed using time- and frequency-domain features. Correlations between audio features and OSA diagnosis/severity were assessed and a predicting model were developed based on these data.

**Results:**

Among the 29 participants with stable COPD, 26 underwent PSG, and 17 were diagnosed with comorbid OSA. Multiple cough and forced exhalation subfeatures correlated significantly with OSA diagnosis and apnea and hypopnea index (AHI). Cough sounds showed the highest correlation with OSA diagnosis (*r* = −0.6629, *p* < 0.001). A logistic regression model using a cough sound subfeature (the median of MFCC_35) achieved 92% accuracy with a Cohen’s kappa value of 0.8276 in predicting OSA in COPD patients.

**Conclusion:**

This study demonstrates a strong association between cough sounds and OSA risk in COPD patients. Cough sounds recorded by smartwatches may serve as a valuable tool for screening OSA in COPD patients, contributing to the management of patients with overlap syndrome.

## 1 Introduction

Chronic obstructive pulmonary disease (COPD) and obstructive sleep apnea (OSA) are both common chronic respiratory diseases with widespread impact. COPD, characterized by irreversible airflow limitation and progressive decline in lung function, most commonly presents with symptoms such as shortness of breath, chronic cough (sometimes with sputum), and fatigue (1, 2). It is one of the leading causes of disease burden, mortality, and healthcare resource consumption worldwide (2). OSA is characterized by recurrent upper airway collapse during sleep, leading to intermittent hypoxia, sleep fragmentation, and sympathetic activation (3). It is closely associated with multiple systemic conditions, including cardiovascular and metabolic diseases, and is often regarded as an upstream risk factor for these disorders (4).

With factors such as population aging, rising obesity rates, air pollution, and persistently high smoking prevalence, the prevalence of both diseases continues to increase (5–8). In 2019, approximately 391.9 million people worldwide were affected by COPD (2, 9), and around 1 billion people were affected by OSA (10). The coexistence of COPD and OSA is referred to as “overlap syndrome (OS)” (11). Overlap syndrome is relatively uncommon in the general population (prevalence: 1.0–3.6%) (12), but its prevalence significantly increases among patients diagnosed with obstructive sleep apnea (prevalence of OS: 7.6–55.7%) or chronic obstructive pulmonary disease (prevalence of OS: 2.9–65.9%) (12, 13).

Patients with OSA-COPD overlap syndrome (OS) typically experience more severe respiratory symptoms and poorer quality of life, with a higher relative risk of exacerbations, hospitalization, and mortality compared to patients with either disease alone (11, 14). Raising awareness, prompt screening, and timely initiating treatment can improve the overall prognosis of OS patients (11, 14). The American Thoracic Society recommends a screening strategy to identify OSA in COPD patients with chronic stable hypercapnia (13).

Therefore, screening and early detection of OSA in COPD patients is of importance. Currently, smart wearable devices are gaining increasing significance in health monitoring and chronic disease management (15). Many studies have also explored the use of smart wearable devices for screening COPD and OSA separately (16, 17). These studies primarily rely on classic physiological parameters, including peripheral oxygen saturation (SpO_2_), respiratory rate (RR), heart rate (HR), heart rate variability (HRV), and activity levels (18–23).

In terms of using audio data for screening COPD, some studies have utilized microphones, smartphones, or dedicated sensors to monitor audio data such as cough sounds for COPD diagnosis and lung function prediction (24–26). Our research team has been using a new smart watch with audio collection capabilities, which collects audio data from cough sounds and breath sounds of COPD patients to assess the severity of COPD (27). When it comes to using audio signals for screening OSA, the most commonly used audio signal is snoring during sleep (28). Until now, there is no research on using cough sounds for screening OSA. If monitoring cough sounds and breath sounds can be used to predict COPD severity while screening for OSA, it would undoubtedly be beneficial for the early identification of OSA in COPD patients, facilitating the management of overlap syndrome (OS).

Thus, we conducted the present study based on our previous research (27), aiming to preliminarily explore the potential value of monitoring daytime cough and forced exhalation sounds for screening OSA.

## 2 Methods

### 2.1 Study population

Patients with suspected chronic obstructive pulmonary disease (COPD) were recruited from the outpatient clinic of the Department of Respiratory and Critical Care Medicine at Peking University First Hospital between June and August 2022. Inclusion criteria required participants to be over 18 years old, in a stable COPD condition, and capable of independently operating a mobile phone. Exclusion criteria included patients with other chronic respiratory conditions, a history of lobectomy or lung transplantation, pleural diseases, severe comorbidities, malnutrition (body mass index, BMI <18 kg/m^2^), bilateral wrist and hand edema or injury, unable to wear a smartwatch. The detailed inclusion and exclusion criteria are provided in our previously published article (27).

All participants provided written informed consent before the study commenced. The study adhered to the ethical guidelines of the Declaration of Helsinki and was approved by the Ethics Committee of Peking University First Hospital (Approval Number: 2022083). It was registered on www.clinicaltrials.gov (NCT05551169).

### 2.2 Clinical examinations

Demographic information such as age, gender, height, and weight was collected, and participants completed questionnaires including the COPD Assessment Test (CAT) and the modified Medical Research Council (mMRC) dyspnea scale. Pulmonary function tests, the 6-min walk test (6MWT), electrocardiogram (ECG), and arterial blood gas analysis were performed. The BODE index was assessed. Overnight polysomnography (PSG) study was conducted.

Based on the results of the pulmonary function tests, COPD patients were classified according to the GOLD criteria: GOLD 1: FEV₁ ≥ 80% of predicted value, GOLD 2: 50% ≤ FEV₁ < 80% of predicted value, GOLD 3: 30% ≤ FEV₁ < 50% of predicted value, and GOLD 4: FEV₁ < 30% of predicted value. Based on the PSG study results, OSA was diagnosed and classified by severity using the apnea-hypopnea index (AHI): AHI ≥ 5 events/h: diagnosis of OSA, AHI ≥ 5 and < 15 events/h: mild OSA, AHI ≥ 15 and <30 events/h: moderate OSA, AHI ≥ 30 events/h: severe OSA.

### 2.3 Wearing the smartwatch and signal recording

Each participant was provided with a smartwatch (Watch GT3/Watch 3, Huawei, China) and was required to wear it continuously for 7–14 days. The device automatically collected photoplethysmography (PPG) and acceleration (ACC) signal data to calculate RR, SpO_2_, HR, and HRV.

Participants were also instructed to record cough and forced exhalation sounds twice daily.

**Figure 1:**
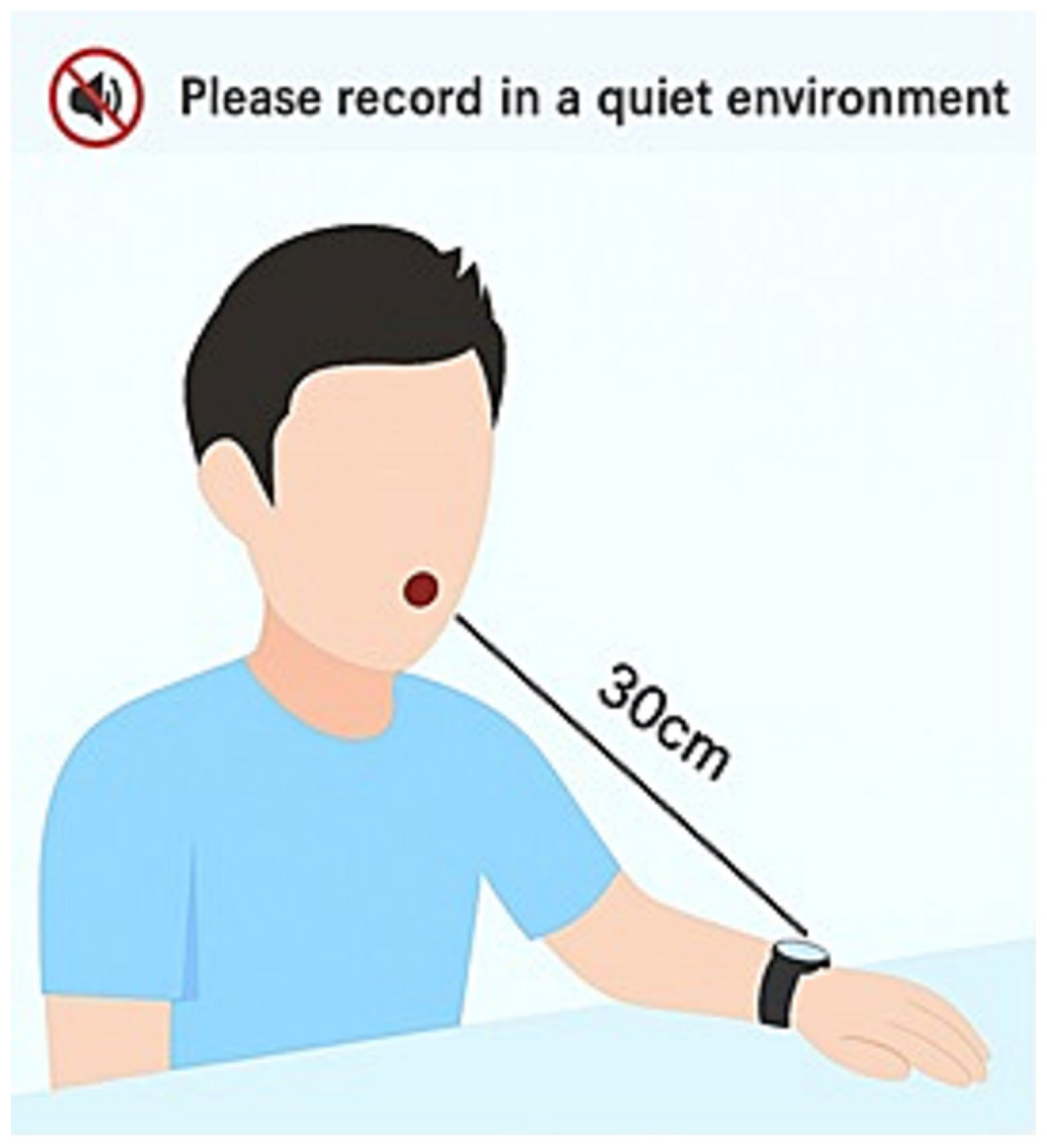
Schematic diagram of the sound recording procedure.

The recordings were conducted in a quiet environment with the participants in a resting state. Subjects maintained a normal seated posture, with their left hand placed flat on the table approximately 30 cm directly in front of the face. For cough sound recording, participants were instructed to slightly open their mouths, take a deep breath, and then cough forcefully two to three times. This procedure was repeated three to five times. For exhalation sound recording, participants took a deep breath and then exhaled as quickly and forcefully as possible through pursed lips, continuing for more than 6 s until no further airflow was expelled. After a 1-min interval, the procedure was repeated at least three times. A schematic diagram is shown in Figure 1. The standardized procedures for data collection are detailed in our previously published article (27).

### 2.4 Signal extraction and data analysis of smartwatch data

#### 2.4.1 Physiological parameters

The photoplethysmography (PPG) and acceleration (ACC) signals collected by the smartwatch were processed using digital signal processing techniques such as filtering to remove noise and extract relevant features. Key physiological parameters including HR, RR, SpO_2_, and HRV were derived.

Data collected during the monitoring period were summarized using statistical indicators such as mean, standard deviation, median, 25th percentile, 75th percentile, and variability measures (including daily and weekly variability). The detailed methods of calculation are described in our previously published article (27).

#### 2.4.2 Audio parameters

The collected audio data were first denoised using speech signal processing techniques. Then, audio segmentation algorithms were applied to extract individual audio segments. For each segment, multidimensional feature extraction methods were used to obtain both time-domain and frequency-domain characteristics. These included commonly used speech signal features such as Mel Frequency Cepstral Coefficients (MFCC), spectral features, chroma (pitch-related) features, time-domain features, and higher-order features. Each type of feature yielded multiple sub-features across different spectrums and audio frames. These sub-features were then aggregated using statistical measures such as mean and variance.

A total of 772 features were extracted from the cough sounds and 1,295 from the forced exhalation sounds. For each participant, all extracted features during the monitoring period were further aggregated using statistical functions including mean, standard deviation, median, 25th percentile, and 75th percentile. As a result, the statistically aggregated cough sound features comprised 3,860 sub-features (772 × 5), and the forced exhalation sound features comprised 6,475 sub-features (1,295 × 5). Detailed values can be found in our previously published article and Supplementary material (27).

### 2.5 Statistical analysis and data processing methods

Count variables were presented as frequencies and percentages. Continuous variables with a normal distribution were expressed as means and standard deviations (SD), while those with a non-normal distribution were expressed as medians and interquartile ranges (IQR). For group comparisons, chi-square tests, *t*-tests and Mann–Whitney *U* tests were used for two-group comparisons. For comparisons among three groups, chi-square tests, analysis of variance (ANOVA), and Kruskal–Wallis *H* tests were applied.

To explore the correlation between smartwatch-derived physiological and audio parameters and clinical data, univariate analysis was first performed. Pearson correlation analysis was used for normally distributed continuous variables, while Spearman correlation analysis was applied to non-normally distributed continuous variables and categorical variables.

**Figure 2:**
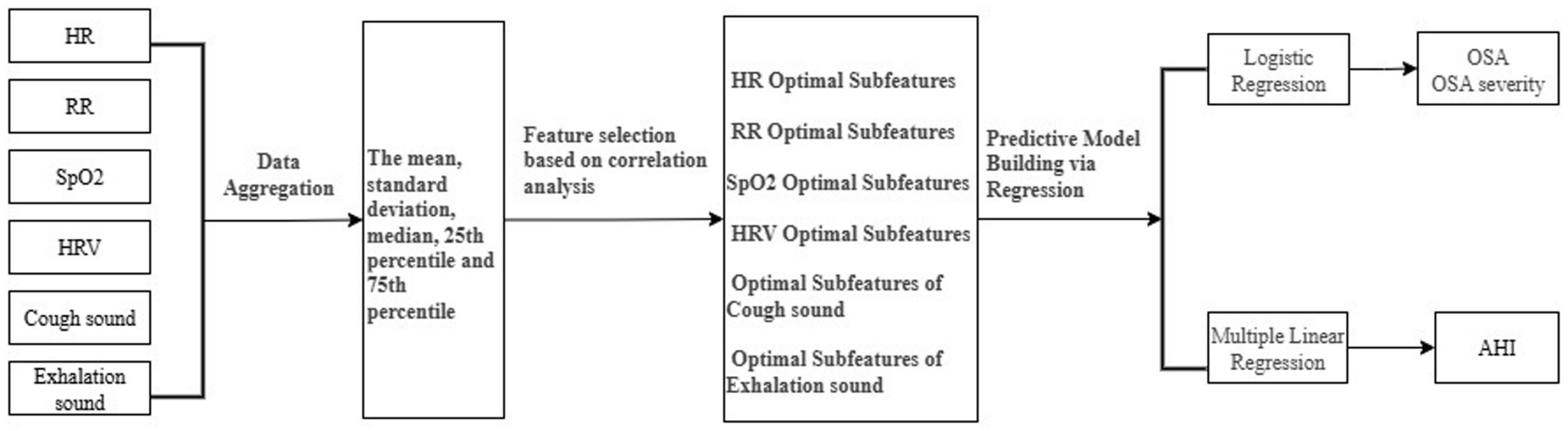
Workflow of data processing.

The subfeature of smartwatch-derived physiological and audio parameters showing the strongest correlation with OSA indicators in the univariate analysis was selected as the representative feature and included in the regression model and predictive models. A logistic regression model was used for the prediction of OSA diagnosis, while a linear regression model was employed for the prediction of AHI. Variable selection was performed using bidirectional stepwise regression combined with the Akaike information criterion (AIC). Multicollinearity was assessed for the variables included in the AIC-selected optimal models, and features with high collinearity were excluded accordingly. Multiple models were compared, and the best-performing model was selected for further analysis. The data processing workflow is illustrated in Figure 2.

All statistical analyses were performed using R version 4.3.2 (R Foundation for Statistical Computing, Vienna, Austria). A *p*-value less than 0.05 was considered statistically significant for all tests.

## 3 Results

### 3.1 Baseline demographics and clinical characteristics

**Figure 3:**
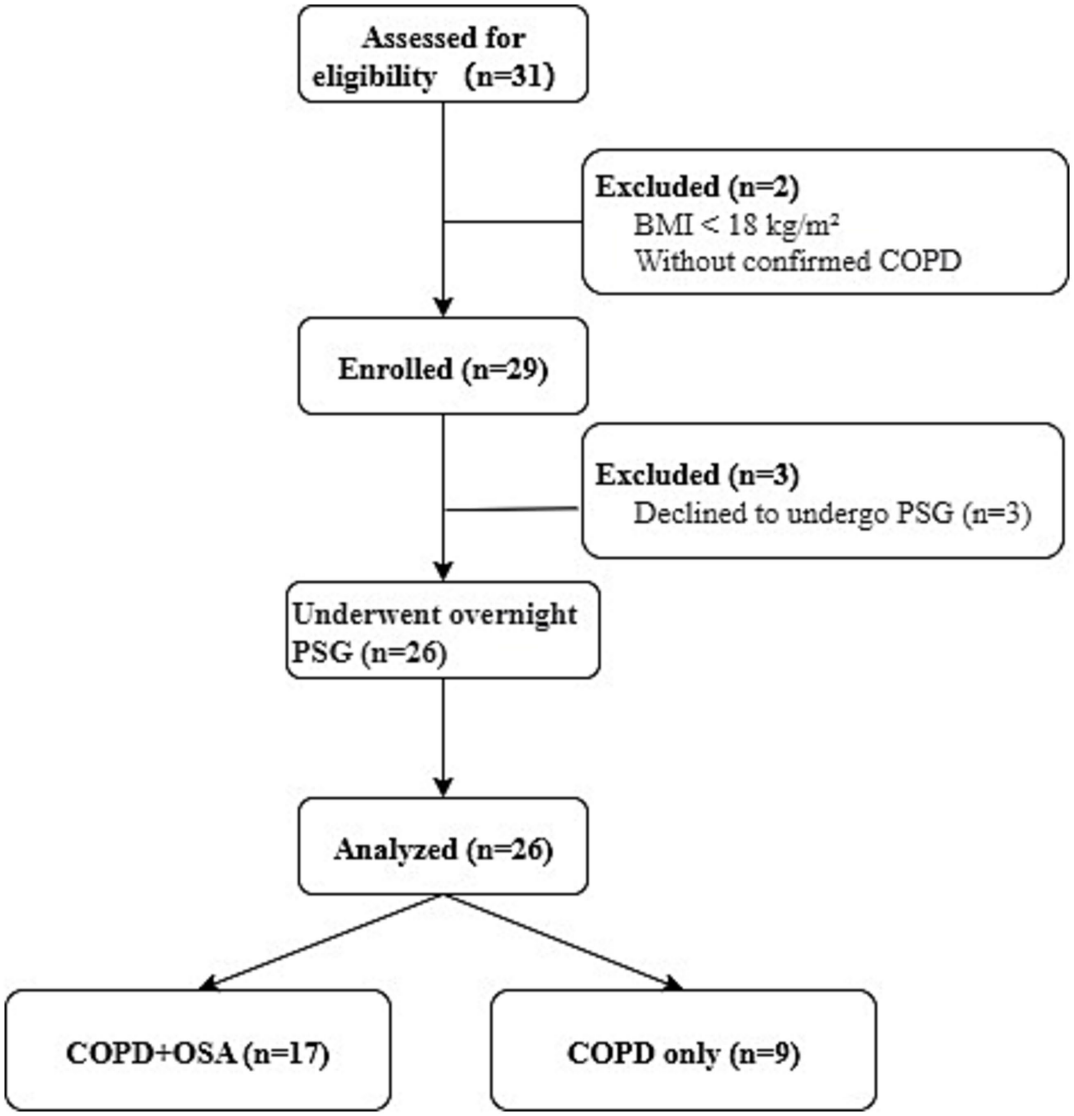
Participant enrollment flow diagram.

A total of 31 patients with suspected COPD were screened, of whom 29 met the inclusion and exclusion criteria and were enrolled in the study. Of these, 26 participants underwent overnight polysomnography (PSG). Based on the PSG results, patients were categorized into two groups: those with comorbid obstructive sleep apnea (COPD-OSA overlap syndrome group, *n* = 17) and those without OSA (COPD group, *n* = 9) (Figure 3).

The COPD-OSA group had significantly higher proportions of male participants, smokers, and higher BMI compared to the COPD-only group (100% vs. 66.7%, 100% vs. 55.6%; 25.8 ± 3.7 vs. 22.5 ± 1.6, *p* < 0.05, respectively). There were no significant differences between the two groups in terms of age, CAT score, mMRC score, BODE index, 6-min walk distance (6MWD), or GOLD classification (*p* > 0.05) (Table 1).

**Table 1:** Characteristics of participants in COPD group and COPD-OSA group.

Characteristic	All (*N* = 26)	COPD (*N* = 9)	COPD-OSA (*N* = 17)	*p-*value
Age (years)	67.2 ± 6.1	68.0 ± 6.0	66.7 ± 6.3	0.617
Sex (male)	23 (88.5)	6 (66.7)	17 (100)	0.032
BMI (kg/m^2^)	24.6 ± 3.5	22.5 ± 1.6	25.8 ± 3.7	0.004
Smoking history				0.008
Never smoked	4 (15.4)	4 (44.4)	0 (0)	
Former/current smoker	22 (84.6)	5 (55.6)	17 (100.0)	
Hypertension	12 (46.2)	3 (33.3)	9 (52.9)	0.429
Cardiovascular disease	3 (11.5)	0 (0)	3 (17.6)	0.529
Diabetes	4 (15.4)	0 (0)	4 (23.5)	0.263
FEV1% (%)	72.6 (50.0, 84.7)	79.7 (34.4, 84.1)	72.3 (59.5, 87.3)^*^	0.419
FVC% (%)	99.2 ± 15.9	100.3 ± 17.9	98.6 ± 15.4	0.795
FEV1/FVC	0.6 (0.5, 0.6)	0.6 (0.3, 0.6)	0.6 (0.5, 0.6)	0.235
Blood gas analysis				
PaO_2_ (mmHg)	84.2 ± 7.8	91.1 ± 7.8	80.8 ± 5.2	0.001
PaCO_2_ (mmHg)	39.8 ± 3.9	38.9 ± 2.5	40.3 ± 4.5	0.411
GOLD grade				0.063
1	10 (38.5)	4 (44.4)	6 (35.3)	
2	10 (38.5)	1 (11.1)	9 (52.9)	
3 + 4	6 (23.1)	4 (44.4)	2 (11.8)	
CAT score	3.0 (2.0, 4.3)	3.0 (1.0, 10.0)	3.0 (2.0, 4.0)	0.847
mMRC score				0.220
0	18 (69.2)	6 (66.7)	12 (70.6)	
1	5 (19.2)	3 (33.3)	2 (11.8)	
≥2	3 (11.5)	0 (0)	3 (17.6)	
BODE index	1.0 (0, 2.0)	1.0 (0, 3.0)	1.0 (0, 1.5)	0.610
BODE group				0.302
0–2	21 (80.8)	6 (66.7)	15 (88.2)	
≥2	5 (19.2)	3 (33.3)	2 (11.8)	
6MWD (m)	448.0 ± 96.5	467.3 ± 82.0	437.6 ± 104.2	0.468
6MWD group				0.628
≥350	21 (80.8)	8 (88.9)	13 (76.5)	
≤349	5 (19.2)	1 (11.1)	4 (23.5)	
SpO_2_ mean	93.5 (92.5, 95.0)	94.3 (93.0, 96.0)	93.2 (92.2, 94.5)	0.099
SpO_2_ lowest	88.0 (83.0, 91.0)	91.0 (88.5, 92.5)	87.0 (80.5, 88.0)	0.001
ODI	8.6 (3.6, 24.8)	1.3 (0.6, 4.1)	19.1 (8.6, 32.5)	<0.001
AHI	8.3 (1.8, 20.6)	1.3 (0.5, 2.6)	17.0 (8.3, 31.1)	<0.001

Data were presented as numbers and percentages [*n* (%)], mean and standard deviation (mean ± SD), median with upper and lower quartiles [median (Q1, Q3)]. BMI, body mass index; FEV1, forced expiratory volume in 1 s; FVC, forced vital capacity; FEV1/FVC, the ratio of FEV1 to FVC; ODI, oxygen desaturation index (≥3% desaturations); AHI, apnea-hypopnea index.

### 3.2 Correlation analysis and predictive modeling of smartwatch physiological parameters and audio data with OSA-related indicators

Physiological parameters collected by the smartwatch, including SpO_2_, RR, HR, and HRV, as well as audio data such as cough and exhalation sounds, were analyzed in relation to OSA diagnosis and AHI. From these six categories of smartwatch data, the most strongly correlated subfeatures are selected as predictive variables to construct predictive models.

#### 3.2.1 Correlation analysis between physiological and audio data with OSA diagnosis, and predictive model development

##### 3.2.1.1 Correlation analysis between physiological and audio data with OSA diagnosis

Using AHI ≥ 5 as the diagnostic criterion, smartwatch-monitored SpO₂, HRV, cough sounds, and exhalation sounds showed significant correlations with OSA diagnosis. RR and HR did not demonstrate significant correlations with OSA diagnosis.

**Figure 4:**
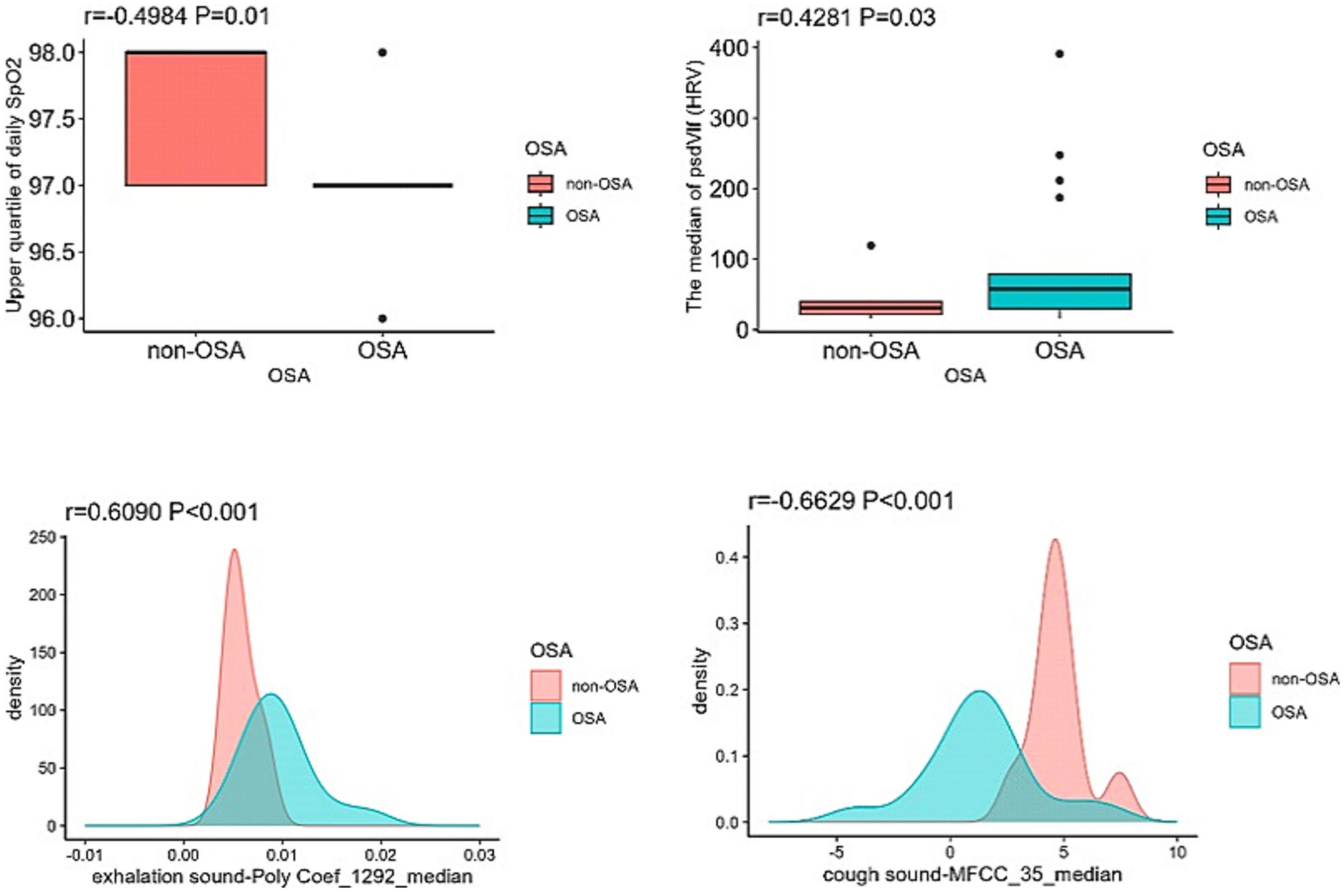
Correlation between smartwatch-derived SpO₂, HRV, exhalation sounds, and cough sounds, as illustrated by boxplots and density plots. In the boxplots, the three lines of the box represent the median, 25th percentile, and 75th percentile, respectively. The black dots indicate outliers. In the density plots, the *x*-axis represents the range of data values, while the *y*-axis indicates the density.

Among these, the subfeatures most strongly associated with OSA diagnosis were as follows: for SpO₂, the upper quartile value during monitoring (all_SpO_2__75%); for HRV, the median power of the very low frequency band in 0–0.04 Hz band (psdVlf_median); for cough sounds, the median value of the Mel-frequency cepstral coefficient (MFCC_35_median) during monitoring; and for exhalation sounds, the median value of the polynomial fitting coefficient (Poly Coef_1292_median) during monitoring. The abbreviations of the subfeatures and their corresponding explanations can be found in Supplementary Table S1. Cough sounds showed the highest correlation (*r* = −0.6629, *p* < 0.001), followed by exhalation sounds (*r* = 0.6090, *p* < 0.001) (Figure 4). Comparisons of these four features between the two groups are presented in Table 2.

**Table 2:** Comparison of smartwatch data between the COPD group and the COPD-OSA group.

Parameters	ALL (*N* = 26)	COPD (*N* = 9)	COPD-OSA (*N* = 17)	*p*
HRV (psdVlf_median)	44.7 (27.4, 78.3)	31.1 (20.0, 40.6)	57.5 (29.5, 132.9)	0.036
SpO_2_ (all_SpO_2__75%)	97.0 (97.0, 98.0)	98.0 (97.0, 98.0)	97.0 (97.0,97.0)	0.013
Exhalation sounds (Poly Coef_1292_median)	0.0083 ± 0.0035	0.0058 ± 0.0015	0.0096 ± 0.0036	0.006
Cough sounds (MFCC_35_median)	2.51 ± 2.68	4.74 ± 1.24	1.33 ± 2.48	0.001

Values are presented as mean ± standard deviation, frequency (percentage), or median (lower quartile, upper quartile).

Poly Coef_1292_median is defined as the median value of the polynomial fitting coefficient of forced exhalation sounds during the monitoring period.

MFCC_35_median refers to the median value of the Mel-frequency cepstral coefficient of cough sounds recorded during the monitoring period.

psdVlf_median refers to the median of psdVlf during the monitoring period. psdVlf is the very low frequency power in the 0–0.04 Hz band.

all_SpO_2__75% refers to the 75th percentile of SpO_2_ during the monitoring period.

##### 3.2.1.2 Predictive model for OSA diagnosis

The above four subfeatures most strongly correlated with OSA diagnosis, HRV (psdVlf_median), exhalation sound (Poly_Coef_1292_median), cough sound (MFCC_35_median), and SpO₂ (all_SpO_2__75%) were initially included as predictor variables in a logistic regression model. After variable selection using the Akaike information criterion (AIC), HRV was excluded, resulting in a refined model that included subfeatures from SpO₂, cough sound, and exhalation sound.

Multicollinearity analysis revealed strong collinearity between these cough and exhalation sound sub-features. To address this, two separate models were constructed: SpO₂ + cough sound, and SpO₂ + exhalation sound. The SpO₂ + cough sound model outperformed the other, with an overall accuracy of 0.88 (95% CI: 0.6878–0.9745), Cohen’s kappa of 0.7492, specificity of 100%, sensitivity of 82.4%, and AUC of 0.897. In comparison, the SpO₂ + exhalation sound model achieved an accuracy of 0.84 (95% CI: 0.6392–0.9546), Cohen’s kappa of 0.6552, specificity of 87.5%, sensitivity of 82.4%, and AUC of 0.890.

**Figure 5:**
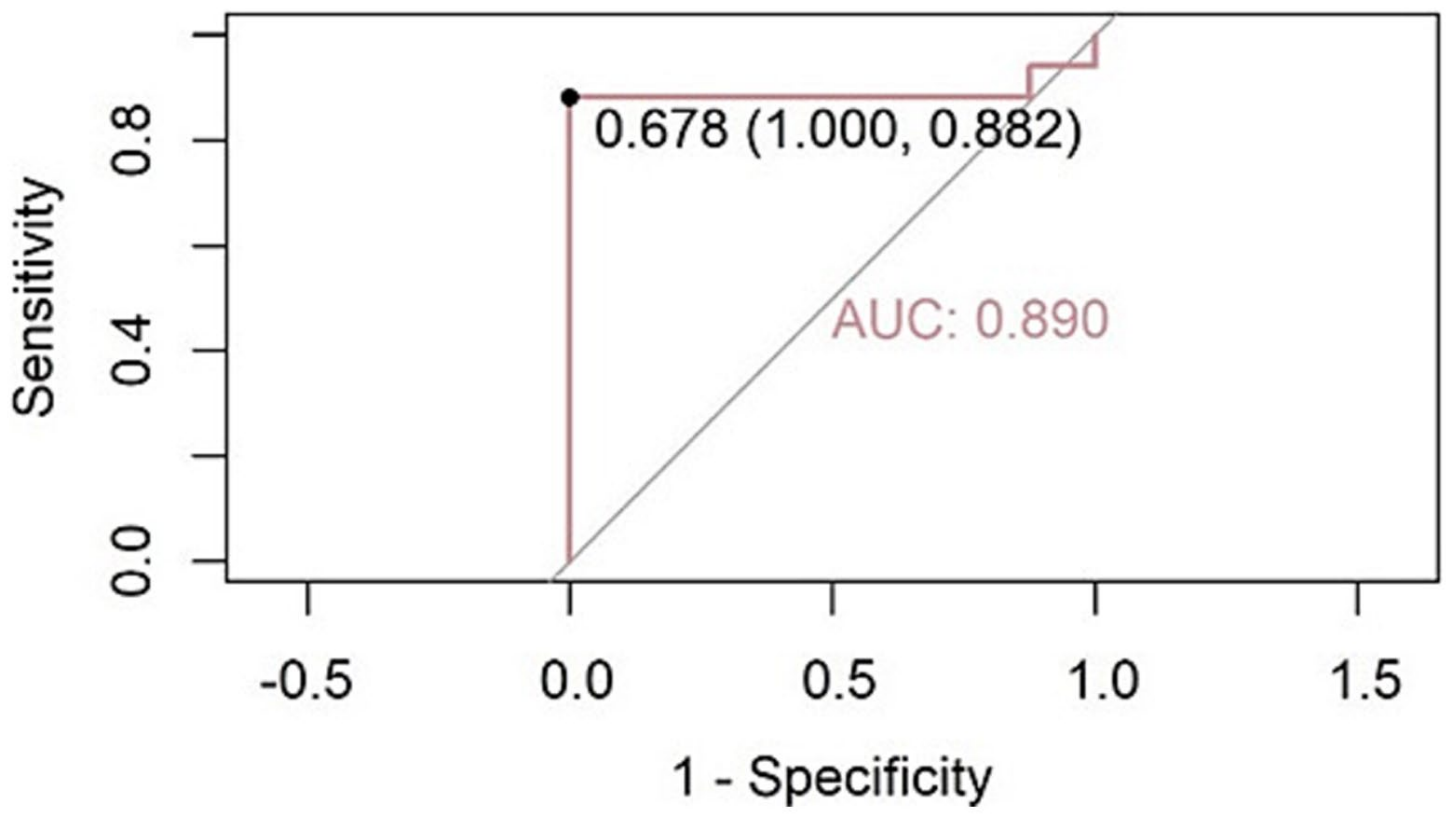
Receiver operating characteristic (ROC) curve for OSA diagnosis using the cough sound feature (MFCC_35_median) as the predictor. The model achieved an AUC of 0.890. At the optimal cutoff threshold of 0.678, the model reached a sensitivity of 88.2% and specificity of 100%.

Thus, the SpO₂ + cough sound model was selected for further analysis. The SpO₂ subfeature (all_SpO_2__75%) was found to be statistically insignificant and was removed. Only the cough sound subfeature (MFCC_35_median) was preserved in the final optimized model. This logistic regression model achieved an accuracy of 92.0%, a sensitivity of 88.2%, a specificity of 100%, an AUC of 0.890, and a Cohen’s kappa value of 0.8276 in diagnosing OSA among COPD patients (Figure 5).

#### 3.2.2 Correlation analysis between physiological and audio data with AHI, and predictive model development

##### 3.2.2.1 Selection of the strongest subfeatures from smartwatch parameters correlated with AHI

Following the above method, the subfeatures of SpO₂, RR, HR, HRV, exhalation sound, and cough sound that showed the strongest correlations with AHI were analyzed. RR and HR showed no significant correlation with AHI (*p* > 0.05, respectively). In contrast, SpO₂, HRV, cough sound, and exhalation sound were significantly correlated with AHI. The subfeatures exhibiting the strongest correlations were as follows: SpO₂ subfeature all_SpO_2__75% (*r* = −0.440, *p* = 0.02), HRV subfeature validRriNum_sd (*r* = −0.541, *p* = 0.01), cough sound subfeature Spectral Contrast_556_sd (*r* = −0.652, *p* < 0.001), and exhalation sound subfeature Poly Coef_1292_median (*r* = 0.664, *p* < 0.001).

##### 3.2.2.2 Predictive model for AHI

**Figure 6:**
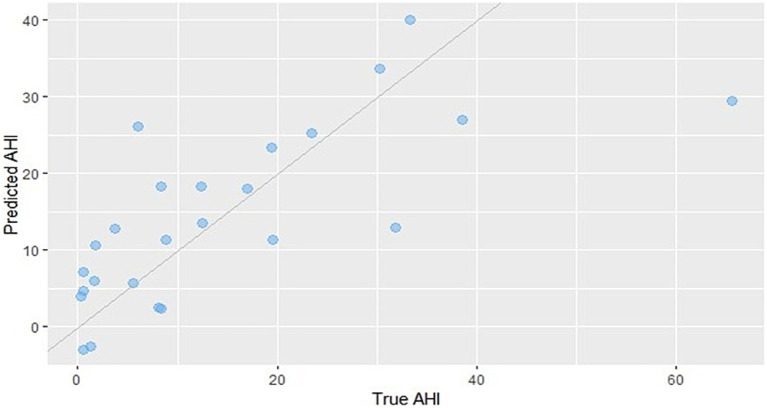
Scatter plot comparing predicted AHI values from the regression model with the actual measured values. The predictive formula is as follows: AHI = 14.364 − 4.017 × HRV + 5.728 × exhalation sound − 4.516 × cough sound.

The above subfeatures most strongly correlated with AHI were used as predictor variables to construct the predictive model. After performing bidirectional stepwise regression and variable selection based on the Akaike information criterion (AIC), the optimal predictive model for AHI was determined. The final model retained HRV (validRriNum_sd), cough sound feature (Spectral_Contrast_556_sd), and exhalation sound feature (Poly_Coef_1292_median). The multiple linear regression equation for predicting AHI is as follows (Figure 6):


$$\begin{array}{l} \mathrm{AHI}=14.364-4.017\times \mathrm{HRV}+5.728\times \text{exhalation sound}\\ -4.516\times \text{cough sound} \end{array}$$

The predictive model achieved a root mean square error (RMSE) of 10.64 events/h, a mean absolute error (MAE) of 7.48 events/h, and an *R*^2^ (coefficient of determination) of 0.5236.

## 4 Discussion

This study demonstrates that cough and forced exhalation sounds in patients with COPD contain signal parameters associated with OSA, indicating potential value for screening and assessment of overlap syndrome. Multiple parameters derived from cough and forced exhalation sounds were found to be related to the diagnosis and AHI. A regression model based on a cough sound subfeature (MFCC_35_median) showed high accuracy in predicting OSA. These findings offer new insights for the early detection of OSA and the management of overlap syndrome in COPD patients.

Identifying coexisting obstructive sleep apnea (OSA) in patients with chronic obstructive pulmonary disease (COPD) holds significant clinical value. The management of patients with overlap syndrome (OS) differs from that of patients with COPD alone. OS patients often present with multiple comorbidities and have a higher risk of developing cardiovascular diseases (CVD) and experiencing acute exacerbations of COPD (29, 30). Moreover, the survival rate of OS patients receiving nocturnal positive airway pressure therapy is significantly lower if they do not receive appropriate treatment (29, 30). The American Thoracic Society recommends a screening strategy to identify OSA in COPD patients with chronic stable hypercapnia (31). Despite its impact, OSA-COPD overlap syndrome has not yet received sufficient attention in clinical practice (11). Early screening and recognition of OSA in COPD patients are of great importance.

As an emerging data source, audio signals have also attracted growing attention in recent years. Compared to traditional physiological signals, audio signals may offer greater specificity and can exhibit stable patterns even in complex environments. Much research has been carried out in this field, and sound signals have come to play an increasingly important role in the monitoring and management of chronic diseases (27, 32, 33).

In recent years, the application of intelligent wearable devices in chronic disease management has become increasingly widespread and rapidly developed (15). With advances in monitoring technology and algorithms, their capabilities have expanded from tracking basic physiological parameters (such as HR, SpO_2_ and physical activity) to new parameters and more complex analyses including sound signals. Previous studies have frequently utilized the built-in microphones of smartphones to collect audio data. For instance, some research has used forced exhalation sounds recorded via smartphone microphones to screen for COPD (34). Similarly, other studies have employed smartphone-based cough sound monitoring for predicting COPD diagnosis, lung function, and airway obstruction (25, 35, 36). Smartwatches, as the most common type of wearable smart device, offer a new avenue for such applications. In our previous study, we initially used a novel smartwatch capable of audio monitoring to collect cough and forced exhalation sounds, demonstrating its potential to assess the severity of COPD (27). However, no studies have yet explored the use of cough and forced exhalation sounds for OSA screening.

In the context of OSA, although direct evidence is currently lacking, several indirect findings and theoretical considerations (37) support the possibility that OSA may influence cough and forced exhalation sounds. Obesity, a major risk factor for OSA, can lead to fat accumulation in the pharyngeal region, which may cause upper airway narrowing and affect the aerodynamic characteristics of airflow—potentially altering the acoustic properties of respiratory sound (38).

In addition, patients with OSA often experience laryngopharyngeal reflux (LPR), which can result in pharyngeal wall edema (39, 40). OSA is also closely associated with chronic cough, and continuous positive airway pressure (CPAP) therapy has been shown to improve chronic cough in OSA patients (40, 41). Chronic cough itself may further contribute to changes in acoustic features such as those of the cough sounds.

In this study, we found that multiple subfeatures of both cough and forced exhalation sounds were associated with OSA diagnosis. Specifically, around 100–200 subfeatures from each sound type showed significant correlations with the diagnosis of OSA. Through regression analysis and the development of the optimal predictive model, we found that the regression model based on cough sound subfeatures demonstrated high accuracy in identifying OSA among COPD patients. These findings highlight the significant screening potential of cough sounds for detecting OSA in patients with COPD.

Given the increasing role of cough sounds monitored by wearable smart devices in the early screening and disease monitoring of COPD patients, utilizing the same signals for both COPD screening and OSA risk assessment would undoubtedly be of significant importance for the detection and management of the overlap syndrome (OS).

In the present study, we used daytime cough sounds to screen for OSA, a condition characterized by respiratory events that primarily occur during sleep. This raises an important question about whether daytime acoustic signals can predict nighttime respiratory events. Previous studies have explored the relationship between daytime speech and the risk of OSA (42–45). These findings suggest the potential utility of wakeful vocal signals as convenient screening tools for assessing the risk and severity of OSA (42–45). However, it is important to note that these methods are currently limited to screening purposes only and cannot replace clinical diagnosis. The use of daytime acoustic signals may offer a more accessible and non-invasive approach to OSA screening.

This study is still in its preliminary exploration stage and has several limitations that need to be addressed in future research. First, the sample size of this study is small, which may introduce sampling bias and affect the stability and representativeness of the results. Therefore, a larger sample size is needed to validate our findings. Second, due to the lack of a normal control group or a pure OSA group, it remains unclear whether the cough sound features identified are specific to COPD-OSA overlap or are common to OSA in general. Therefore, our conclusions are limited to the COPD population and cannot be extrapolated beyond this group. Third, differences in microphone sensitivity, sampling rate, and built-in noise reduction algorithms may exist across devices. The current results are primarily based on the devices used in this study. Future research should validate the model’s generalizability and robustness in various smart devices. Last but not least, due to the exploratory nature of this study and in order to record audio data more clearly and accurately, we chose voluntary coughs. However, spontaneous coughs may better reflect the patient’s actual pathological state.

In summary, this study innovatively proposes using a smartwatch to collect cough and forced exhalation sounds to assess the risk of OSA in COPD patients. This method provides a new perspective on traditional sleep apnea monitoring with wearable devices, which usually involves the use of pulse oximetry and snoring sounds.

Given the widespread use of wearable devices in home-based chronic disease management and the common occurrence of cough as a symptom in chronic respiratory conditions, this method can help in the early identification of OSA in patients with existing chronic airway inflammatory diseases. In future studies, in addition to expanding the sample size and including healthy controls and general OSA patients as comparisons, it would be valuable to explore the use of spontaneous cough audio features for OSA screening, in addition to voluntary cough. Furthermore, the mechanisms linking OSA and cough sounds remain to be explored in greater depth through basic research.

## Data Availability

The raw data supporting the conclusions of this article will be made available by the authors, without undue reservation.
