# Supplementary material for: The value of cough sound monitoring via an audio-enabled smartwatch for OSA screening in COPD patients: a cross-sectional exploratory study
Source: Front Med (Lausanne). 2025 Oct 8;12:1650014. doi: 10.3389/fmed.2025.1650014 (PMC12540090; doi:10.3389/fmed.2025.1650014)
Supplement: Supplementary file 1 [file Table_1.DOCX]

Table s1: The abbreviations of the subfeatures and their corresponding description

| Parameters | Sub-feature | Description |
| --- | --- | --- |
| SpO2 | allSpO_2_75 | The 75th percentile of SpO2 during the monitoring period |
| HRV | psdVlf_median | The mean of psdVlf during the monitoring period. psdVlf is the very low frequency power in the 0-0.04 Hz band |
| HRV | validRriNum_sd | The standard deviation of validRriNum. validRriNum is defined as the number of valid RR intervals during sleep. |
| cough sounds | MFCC_35_median | The median of MFCC_35 during the monitoring period. MFCC(Mel-Frequency Cepstral Coefficient). It represents the distribution of the energy of the audio in different frequency ranges |
| cough sounds | Spectral Contrast_556_sd | The standard deviation of Spectral Contrast. |
| exhalation sounds | Ploy Coef_1292_median | The median of Poly Coef_1291.Poly Coef(Polynomial fitting coefficients) are the coefficients obtained by fitting an n-order polynomial to the corresponding values in the spectrogram. |
